# Suffer or Survive: Decoding Salt-Sensitivity of Lemongrass and Its Implication on Essential Oil Productivity

**DOI:** 10.3389/fpls.2022.903954

**Published:** 2022-06-09

**Authors:** Mohammad Mukarram, M. Masroor A. Khan, Andleeb Zehra, Peter Petrik, Daniel Kurjak

**Affiliations:** ^1^Advanced Plant Physiology Section, Department of Botany, Aligarh Muslim University, Aligarh, India; ^2^Department of Integrated Forest and Landscape Protection, Faculty of Forestry, Technical University in Zvolen, Zvolen, Slovakia; ^3^Global Change Research Institute, Czech Academy of Sciences, Brno, Czechia

**Keywords:** antioxidants, *Cymbopogon*, essential oil, geraniol dehydrogenase, medicinal and aromatic plant (MAP), oxidative stress, ROS, salinity

## Abstract

The cultivation of lemongrass (*Cymbopogon flexuosus*) crop is dominated by its medicinal, food preservative, and cosmetic demands. The growing economy of the lemongrass market suggests the immense commercial potential of lemongrass and its essential oil. Nevertheless, the continuous increase of the saline regime threatens the growth and productivity of most of the plant life worldwide. In this regard, the present experiment explores the salt sensitiveness of the lemongrass crop against five different levels of salt stress. Metabolomic analyses suggest that lemongrass plants can effectively tolerate a salt concentration of up to 80 mM and retain most of their growth and productivity. However, extreme NaCl concentrations (≥160 mM) inflicted significant (α = 0.05) damage to the plant physiology and exhausted the lemongrass antioxidative defence system. Therefore, the highest NaCl concentration (240 mM) minimised plant height, chlorophyll fluorescence, and essential oil production by up to 50, 27, and 45%. The overall data along with the salt implications on photosynthetic machinery and ROS metabolism suggest that lemongrass can be considered a moderately sensitive crop to salt stress. The study, sensu lato, can be used in reclaiming moderately saline lands with lemongrass cultivation converting such lands from economic liability to economic asset.

## Introduction

Lemongrass (*Cymbopogon flexuosus*) is a C_4_ perennial cash crop belonging to the Poaceae family and is commonly known as East Indian or Malabar grass. The major share of lemongrass cultivation is based on its essential oil production. The lemongrass essential oil (LEO) has enormous medicinal and commercial potential. The pharmacological benefits of LEO are derived from its antimicrobial, anticancer, insect-repellent, and antioxidant properties (Mukarram et al., 2021a,b). These bioactivities are further exploited in cosmetics and food packaging and safety. Lemongrass comprises a rich source of vitamins (A, C, E, folate, niacin, and riboflavin), protein, and mineral nutrients (Fe, Mn, Zn, Ca, N, P, K) making it an excellent nutritional source (Aftab et al., 2011; Gaba et al., 2020). The high cellulosic (cellulose, 39.5%; hemicellulose, 22.6%) structure of lemongrass along with lignin (28.5%) abundance make it a suitable candidate for the synthesis of vitamin A, β- and methyl-ionones, paper, pulp, silica, and composites (Haque et al., 2018). Few recent studies suggested that lemongrass can also be used to produce biofuel (Dhinesh et al., 2016; Sathiyamoorthi and Sankaranarayanan, 2016; Venkatesan et al., 2019). The perspective research into these lesser developed sectors can further augment the importance of lemongrass plants. The immense benefits of lemongrass and its essential oil are nudging crop scientists to develop new sustainable methods of enhancing lemongrass growth and productivity (Mukarram et al., 2021c,d).

On a global scale, the lemongrass is harvested over 16,000 ha of land which corresponds to an annual production of 1000 t of LEO (Haque et al., 2018). Mukarram et al. (2021b) reviewed the lemongrass economics and suggested a rapid growing pattern in India and globally as well. It was suggested that the estimated LEO market in 2019 was 247 million US$ which will be approximately doubled by 2027. Moreover, the Indian export of lemongrass was escalated with more than 1250% growth in the past two decades (2001–2020). Nevertheless, considering the soaring pattern, the demand for lemongrass and its essential oil is expected to be intensified in the upcoming years.

Massive population growth and urbanisation are discouraging agricultural lands and degrading the soil. Soil salinity is one of the leading threats to agricultural production worldwide. More than 800 million hectares are said to be salt-affected, which accounts for about 27 billion US$ global economic loss each year (Qadir et al., 2014). Soil is called saline when its electrical conductivity exceeds or equals 4 dS m^–1^ which is equivalent to 40 mM of NaCl (USSL, 2005). While primary salinity is naturally occurring, secondary salinity is largely contributed by anthropogenic activities including improper soil clearing and irrigational practices (Pitman and Läuchli, 2002; Munns, 2005). Most crops are glycophytes and cannot grow well in high salt concentrations. Thus, depending on the severity, salinity can regulate seed germination, cell expansion, stomatal conductance, photosynthesis, and other metabolic and developmental pathways (Shabala and Cuin, 2008; Machado and Serralheiro, 2017). In such crops, salinity can inflict moderate to acute harm depending on certain factors such as salt concentration and plant resilience (Munns and Tester, 2008). The *modus operandi* of salinity stress occurs in two phases: The first phase imposes osmotic stress on plants by restricting the water uptake that mimics drought-like situations (Munns, 2002). The osmotic phase distorts the ion homoeostasis and builds ion excess in the plant (ionic stress phase) in a salt-specific effect (van Zelm et al., 2020). These primary implications endorse a more comprehensive range of secondary effects induced by the saline regime such as oxidative stress and nutritional imbalance (Negrão et al., 2017). The ion-excess effect arises when salt concentration hits toxic levels inside the leaves. Therefore, the plants first try to minimise the salt uptake through roots and later separate the remaining at the cellular and tissue level to evade excessive salt accumulation in the cytoplasm of photosynthetic leaves (Munns, 2005; Flowers and Colmer, 2008). The osmotic stress and ionic stress support the overproduction of various reactive species that contributes to oxidative stress. Although reactive oxygen species (ROS) are produced as a by-product of many physiological processes including photosynthesis and respiration under the normal environment that can facilitate plant signalling, their overproduction during abiotic stress poses greater oxidative stress that damages proteins, lipids, and nucleic acids and limits crop growth and yield (Ashraf, 2009; Mukarram et al., 2021e). Similar salt-induced restrictions on plant development and productivity were observed in many crops of economic importance including rice, wheat, barley, and maize (Zeng and Shannon, 2000; Munns et al., 2006; Widodo et al., 2009; Zörb et al., 2019; Mukarram et al., 2021f).

Keeping in the mind the decreasing agricultural landholdings and the growing demand for lemongrass and its oil, we conducted the present experiment to assess the salt-sensitivity of the lemongrass crop. We hypothesised that the lemongrass, being a member of the grass family, would withstand the low (40 mM NaCl) to moderate (80 mM NaCl) salinity. The present experiment is an attempt to suggest to the wider audience the cultivation of lemongrass crops in lands deemed unsuitable for traditional cereal crops due to soil salinity. Furthermore, though lemongrass has a well-defined defence system comprising enzymatic and non-enzymatic antioxidants (Aziz et al., 2014) that might counter salinity-induced oxidative stress, it would be interesting to observe the potential of this defence system against severe salinity (≥160 mM).

## Materials and Methods

### Plant Material and Growth Conditions

The present study was performed at the net house of the Department of Botany, Aligarh Muslim University, Aligarh, India (27°52′ N, 78°51′ E, 187 m a.s.l.). The lemongrass [*Cymbopogon flexuosus* (Steud.) Wats var. Nima] was propagated from healthy slips brought from Central Institute for Medicinal and Aromatic Plants, Lucknow, India. The slips of the plants were sterilised with 0.2% HgCl_2_ and washed afterward repetitively. The plant slips were transferred to the 7 L capacity earthen pots (25 cm × 25 cm) mixed with sand, clay, and peat in a 7/2/1 ratio of their weights. Plants were irrigated with 250 mL of double-distilled water (DDW) daily. The average temperature and humidity were recorded as 17–25°C (±4°C), and 68% (±5%) during the experimental timeline. Soil evaluation at the Soil Testing Laboratory of Indian Agriculture Research Institute (IARI), New Delhi, India, described the soil physical texture as sandy loam while chemical variables were as follows: pH, 7.6; electrical conductivity, 0.52 dS m^–1^; N, 94.8 mg kg^–1^ of soil; P, 8.9 mg kg^–1^ of soil; K, 136.5 mg kg^–1^ of soil.

Plants were cultivated after 30 days from the final salt treatment for all the morpho-physiological assessments. The experimental setup was arranged in a complete random block design. Three lemongrass plants were grown in each pot, and each pot was considered as one replicate. Each treatment was replicated three times.

### Induction of Salinity Stress

Five different salt regimes (0, 40, 80, 160, and 240 mM) were created for the present study (Table 1). A salt concentration of 40 mM NaCl was provided to the lemongrass plants on alternate days to induce these saline regimes. Thus, it took 1 day to attain required salt concentration for Treatment 2 (40 mM NaCl), 3 days for Treatment 3 (80 mM NaCl), 7 days for Treatment 4 (160 mM NaCl), and 11 days for Treatment 5 (240 mM NaCl). To maintain uniform salinity the above salt concentration was given in the form of an aqueous solution (40 mL). If the salt stress is added in one go, the plant might not grow at all and could take considerable time to recover, depending on the level of osmotic stress it caused. Therefore, salt treatments in the present study were added gradually to attain the required NaCl level and prevent the plant from having osmotic shock.

**TABLE 1:** Different salt regimes generated during the present experiment for assessing salt tolerance in lemongrass.

Salt regime	Equivalent electrical conductivity	NaCl concentration
Non-saline	0 dS m^–1^	0 mM
Slightly saline	4 dS m^–1^	40 mM
Moderately saline	8 dS m^–1^	80 mM
Highly saline	16 dS m^–1^	160 mM
Extremely saline	>16 dS m^–1^	240 mM

The salt conditions were devised based on the classification provided by the US Salinity Laboratory (Richard, 1954) to gauge the salinity tolerance level of lemongrass. Therefore, the present study consisted following salt regimes.

**FIGURE 1:**
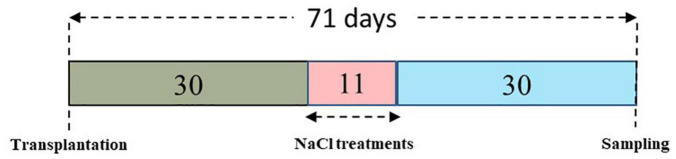
The experimental timeline for the present study.

Figure 1 illustrates all the major and significant events in the experimental timeline, from the growing of the lemongrass plants to their harvesting.

### Quantification of Photosynthetic Fitness

The chlorophyll content was determined from the completely expanded lemongrass leaves by using the Minolta chlorophyll metre (SPAD-502, Konica Minolta Sensing Inc., Osaka, Japan).

Chlorophyll fluorescence (Fv/Fm) was expressed as the quantum efficiency of open photosystem II (PSII) centres, which were dark-adapted for 30 min before measurement. Fv/Fm was determined in the daytime on the adaxial surface of the first fully developed leaf by a chlorophyll fluorometer, PAM-2000 (Walz, Effeltrich, Germany).

### Gas Exchange

The gas exchange between the plant and the external environment was determined in terms of net photosynthetic rate (P_*N*_), stomatal conductance (g_*s*_), and transpiration rate (E). All the variables were quantified in the first expanded plant leaf through an Infra-red Gas Analyser (LI-COR 6200, Portable Photosynthesis System, NA, United States). The leaf area for the assessment was 6 cm^2^. The gas exchange was analysed at light-saturating intensity (PAR: 780–800 μmol m^–2^ s^–1^) with an air temperature of 25°C, relative humidity 65–85%, and 370 ± 5 μmol mol^–1^ atmospheric CO_2_ concentration.

### Markers for Oxidative Damage Assessment

#### Thiobarbituric Acid Reactive Substances Content

The TBARS concentration was assessed to determine the lipid peroxidation in lemongrass leaves according to Cakmak and Horst (1991). Fresh lemongrass leaves (0.5 g) were ground in trichloroacetic acid (TCA, 5 ml, 0.1% w/v) followed by centrifugation (12,000 × *g*, 5 min). The supernatant (1 mL) along with tetrabutylammonium (4 mL, 0.5% w/v) was mixed with TCA (20% w/v) and incubated (90°C, 30 min) followed by ice bath treatment and a second round of centrifugation (10,000 × *g*, 5 min). The TBARS content was calculated as malondialdehyde equivalents by noting the absorbance of the mixture spectrophotometrically (Shimadzu UV-1700, Tokyo, Japan) at 532 nm followed by the subtraction of the absorbance recorded at 600 nm for further correction of non-specific turbidity.

#### Hydrogen Peroxide Content

The H_2_O_2_ content was calculated through a peroxidase-dependent essay as developed by Okuda et al. (1991) to estimate the oxidative imbalance in the salt-stressed lemongrass. The plant leaves were sampled and ground in perchloric acid. The subsequent solution was centrifuged and blended with peroxidase to commence the reaction. The optical density of the reaction mixture was noted for 3 min at 590 nm.

### Lemongrass Defence System

#### Preparation of Enzyme Extract

The lemongrass leaves were cut and frozen with liquid nitrogen. The frozen leaves were crushed using a mortar and pestle into a powder which was mixed with an extraction solution (5 mL w/v) comprising potassium phosphate buffer (100 mM, pH 7.8), polyvinylpyrrolidone (1% w/v), and triton-X-100 (0.5% v/v). Afterward, the mixture was centrifuged (15,000 × *g*, 5 min, 4°C) and the supernatant was utilised to distinguish various antioxidants activities (Kuo et al., 1982).

##### Superoxide Dismutase Activity

The methods as developed by Beauchamp and Fridovich (1971) were followed to mark SOD (E.C. 1.15.1.1) activity. A reaction mixture (4 mL) of enzyme extract (0.1 mL) with riboflavin (1 mM), methionine (9.9 mM), triton-X-100 (0.02%), nitro blue tetrazolium (NBT, 55 mM), and EDTA (2 mM) was illuminated and sustained (30°C, 1 h). Later, the absorbance was noted at 560 nm and SOD activity was expressed as the amount needed to half inhibit the NBT reaction.

##### Catalase Activity

The CAT (E.C. 1.11.1.6) activity was assessed following a similar procedure as explained in our previous report (Mukarram et al., 2021d). A reaction mixture with enzyme extract (0.04 mL), potassium phosphate buffer (2.6 mL, 50 mM, pH 7), and H_2_O_2_ (0.4 mL, 15 mM) was prepared and centrifuged (12,500 × *g*, 20 min, 4°C). Later, CAT activity was calculated from H_2_O_2_ disappearance at 240 nm.

##### Peroxidase Activity

The activity of the POX (E.C. 1.11.1.7) enzyme was detected according to Kumar and Khan (1982). The enzymatic activity for POX was expressed as the conversion rate of pyrogallol to purpurogallin at 420 nm.

#### Proline Content

The osmolyte proline was quantified in lemongrass leaves using an earlier protocol (Bates et al., 1973). Fresh leaves (0.25 g) were chopped in sulfosalicylic acid (2.5 mL, 3%) and centrifuged (10,000 × *g*, 10 min), followed by water-bath boiling (100°C, 1 h) with a mixture of sulfosalicylic acid (2.5 mL), glacial acetic acid (1 mL), and acid ninhydrin solution (1 mL). The addition of toluene (3 mL) initiated chromophore generation whose absorbance was noted at 520 nm for proline estimation.

### Determination of Growth Variables

The plant growth was assessed on three variables, i.e., plant height, dry weight, and leaf area. The dry weight was determined after drying the plant for 24 h in a hot-air oven at 80°C. The dried plants were then weighed using an electric balance.

### Essential Oil Machinery

#### Geraniol Dehydrogenase Activity

Geraniol dehydrogenase (E.C. 1.1.1.183) is a key enzyme in essential oil biosynthesis in lemongrass plants. To address the impact of salinity on lemongrass oil biosynthesis machinery, GeDH activity was determined as described in our earlier experiment (Mukarram et al., 2021c). A reaction mixture (pH 7.5) was prepared by grounding lemongrass leaf tissue in tricine-NaOH, Polyclar AT, glycerol, and β-mercaptoethanol, thiourea. The geraniol dependent-NADP^+^ conversion was used as a marker for GeDH activity and was expressed in n katal mg^–1^ protein.

#### Essential Oil Productivity

The essential oil production was determined by the gravimetrical method developed by Guenther (1972). Fresh lemongrass leaves (100 g) were cut into tiny pieces and transferred to a flask attached to the Clevenger’s apparatus (Borosil, India). The cut leaves were boiled in DDW in the flask for 3 h using a heating mantle. The vapour formed contained the essential oil mixed with DDW. This vapour was cooled down using a condenser and captured into the receiver.

### Statistical Analyses and Graphical Presentations

**FIGURE 2:**
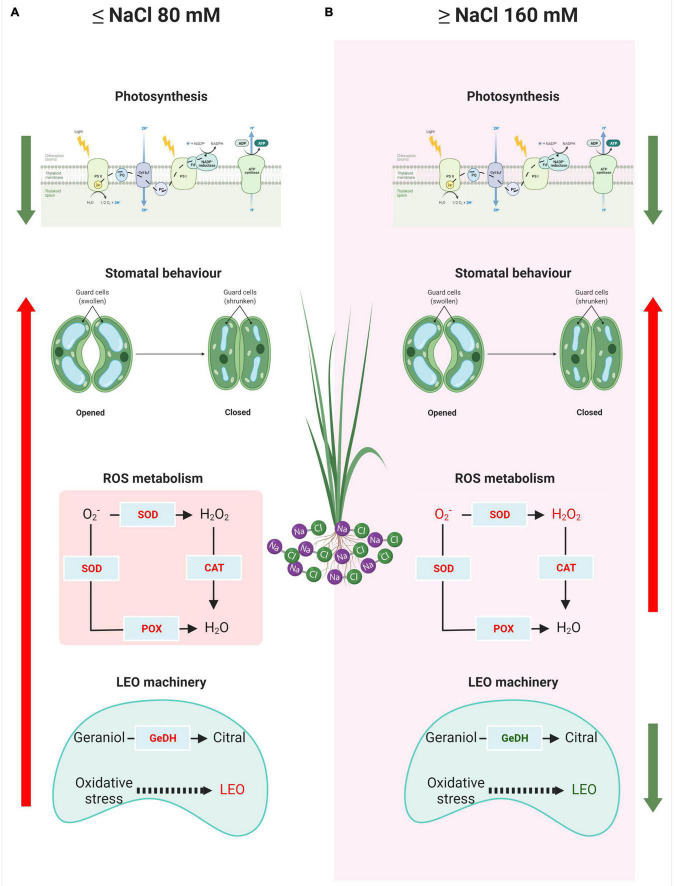
Dissimilar response model of a lemongrass plant at low **(A)** and high **(B)** salt concentration. The upward arrows indicate upregulation while green downward arrows signify downregulation in the process. O_2_^–^, superoxide ion; H_2_O_2_, hydrogen peroxide content; CAT, catalase activity; POX, peroxidase activity; SOD, superoxide dismutase activity; GeDH, geraniol dehydrogenase activity; LEO, lemongrass essential oil.

The normal distribution of the data was first tested for each treatment by the Shapiro–Wilk test. The homogeneity of variance among treatments was tested with Bartlett’s test. One-way analysis of variance (ANOVA) was used to test the salt effect on lemongrass growth, development, and productivity. Duncan’s multiple range *post hoc* test was used to determine the significance of differences among the treatments. Correlation analysis was used to analyse relationships between all parameters observed for control and four stress treatments. Principal component analysis was applied to measured parameters to explore the overall relationship between them and examine the positioning of the treatments within this system. All statistical analyses were conducted at the replicate level and α = 0.05 in SPSS-25.0 for Windows (SPSS, Inc., Chicago, IL, United States). Figure 2 was created with BioRender.com.

## Experimental Results

### Lemongrass Growth and Development Under Salt Stress

**FIGURE 3:**
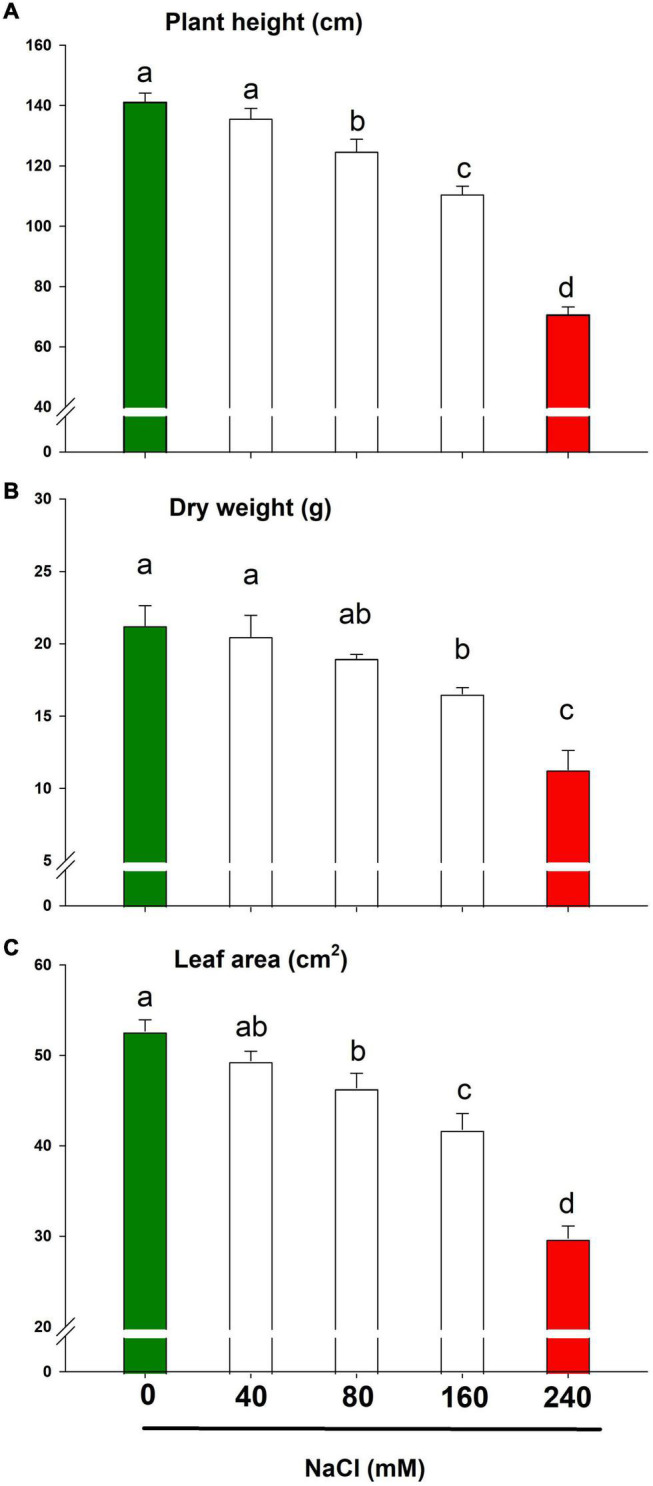
Growth parameters of lemongrass plant under the different salt regimes (0, 40, 80, 160, and 240 mM NaCl) depicting plant height **(A)**, dry weight **(B)**, and leaf area **(C)**. Each bar represents mean ± SE (*n* = 3). Means followed by the same letter(s) do not differ by LSD test at 5% probability level (α = 0.05). The colors are just visual ques for readers to quickly distinguish between control and most stressed treatment.

Lemongrass growth and development were compromised during all the salt concentrations. However, under slightly to moderate salinity (≤80 mM), lemongrass plants retained most of their height, biomass, and leaf area as compared to control plants (Figures 3A–C). The lemongrass retained about 89% of its height under moderate salinity whereas this percentage was observed to be about 90 and 88 for dry weight and leaf area measurements, respectively. Nevertheless, at the advent of the highly saline regime, plant height was reduced by 21% over the control. Similarly, dry weight and leaf area were restricted by 22 and 20% over the control under NaCl 160 mM. The maximised reduction in all the studied variables was observed with NaCl 240 mM including plant height (50%), dry weight (47%), and leaf area (43%) when compared with the control.

### Photosynthetic Machinery During Salinity

**FIGURE 4:**
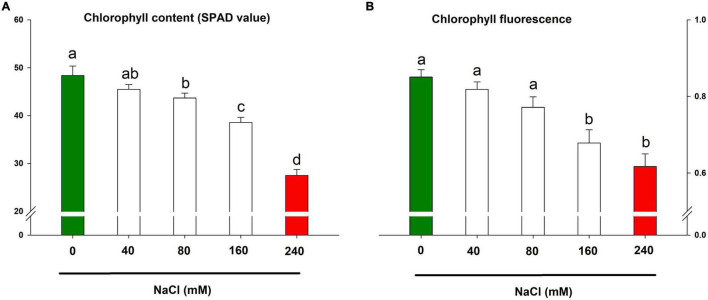
Impact of NaCl concentrations (0, 40, 80, 160, and 240 mM) on chlorophyll content **(A)** and maximum quantum efficiency of PSII photochemistry **(B)** of lemongrass plants. Each bar represents mean ± SE (*n* = 3). Means followed by the same letter(s) do not differ by LSD test at 5% probability level (α = 0.05). The colors are just visual ques for readers to quickly distinguish between control and most stressed treatment.

The chlorophyll content and chlorophyll fluorescence are important markers for the photosynthetic health of a plant. In the present study, the retarded growth traits were underpinned by collapsed photosynthesis in lemongrass leaves. The result showed significant downregulation in photosynthetic pigment and fluorescence with each salt concentration (Figures 4A,B). Nevertheless, chlorophyll content was diminished by about 43% over the control by NaCl 240 mM. The same treatment caused the maximised distortion of 27% in chlorophyll fluorescence over the control plants.

### Lemongrass Gas Exchange and Salt Stress

**FIGURE 5:**
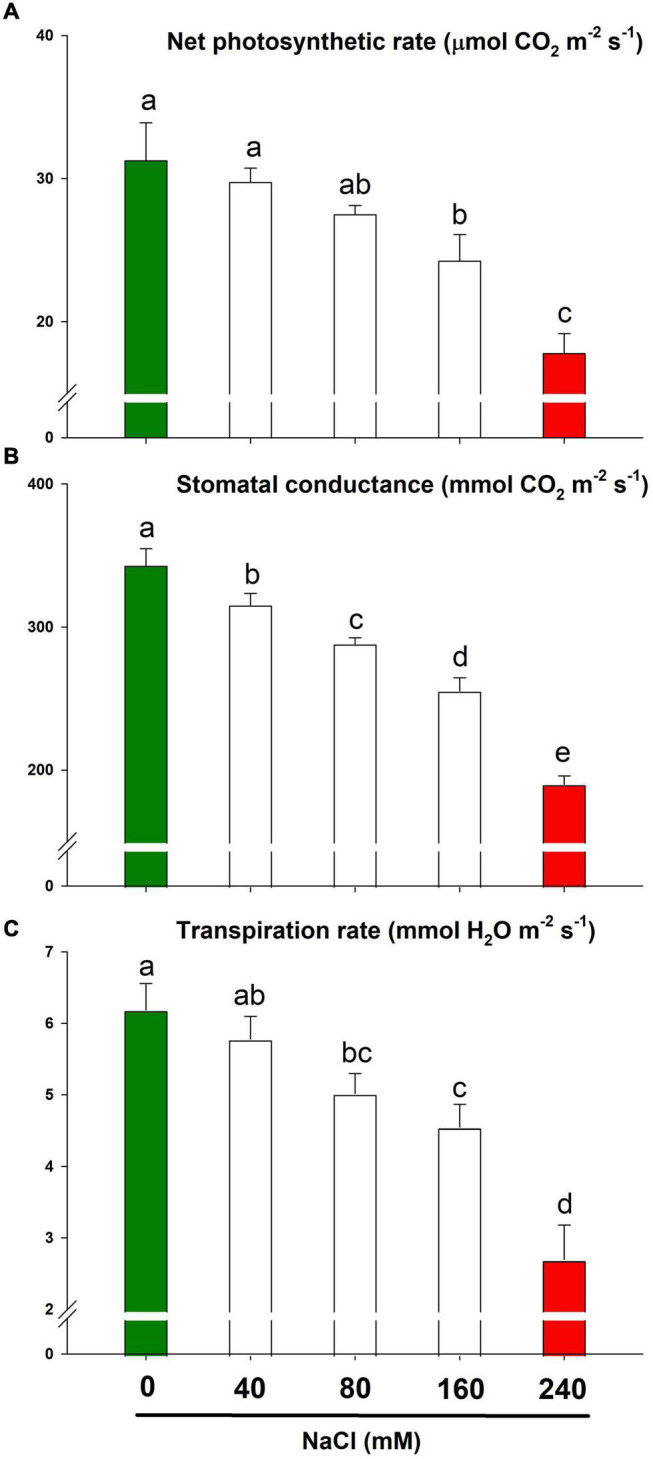
The gas exchange response of lemongrass to NaCl concentrations (0, 40, 80, 160, and 240 mM) with the focus on net photosynthetic rate **(A)**, stomatal conductance **(B)**, and transpiration rate **(C)**. Each bar represents mean ± SE (*n* = 3). Means followed by the same letter(s) do not differ by LSD test at 5% probability level (α = 0.05). The colors are just visual ques for readers to quickly distinguish between control and most stressed treatment.

Lemongrass gas exchange was determined in terms of net photosynthetic rate, stomatal conductance, and transpiration rate. Although a slightly saline regime (NaCl 40 mM) did not bring any significant (α = 0.05) changes in the net photosynthetic and transpiration rate, higher salt content restricted stomatal behaviour to a minimum (Figures 5A–C). The extremely saline regime, i.e., NaCl 240 mM limited P_*N*_ by about 43% over the control while g_*s*_ was reduced by about 45%. Similarly, the values for E were reduced by 27 and 56% with 160 mM and 240 mM of NaCl, respectively.

### Salt-Induced Oxidative Damage

**FIGURE 6:**
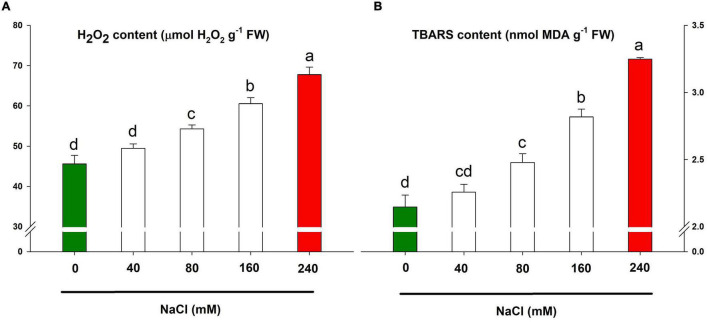
Oxidative stress markers for assessing the salt-sensitivity in lemongrass plants. H_2_O_2_ content **(A)** and TBARS content **(B)** are depicted here. Each bar represents mean ± SE (*n* = 3). Means followed by the same letter(s) do not differ by LSD test at 5% probability level (α = 0.05). The colors are just visual ques for readers to quickly distinguish between control and most stressed treatment.

The H_2_O_2_ and TBARS contents are the known marker for oxidative stress and lipid peroxidation and thus, in turn, cellular damage. Both H_2_O_2_ and TBARS contents were positively correlated with increasing salt concentrations allowing aggravated oxidative stress and lipid peroxidation with higher doses, e.g., 160 mM and 240 mM (Figures 6A,B). The highest accumulation of H_2_O_2_ and TBARS contents was caused by NaCl 240 mM application loosely followed by NaCl 160 mM.

### Lemongrass Defence System for Salt Tolerance

**FIGURE 7:**
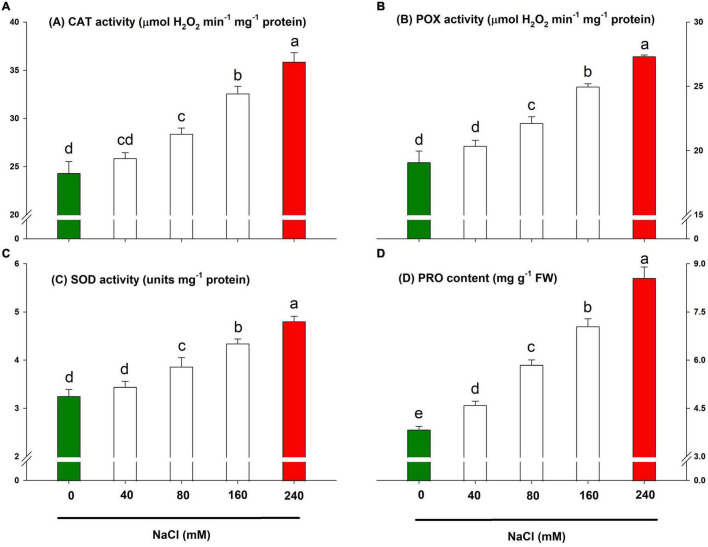
Lemongrass defence system and salinity stress. The activities of enzymatic antioxidants such as catalase **(A)**, peroxidase **(B)**, and superoxide dismutase **(C)**, and the content of osmolyte proline **(D)** are depicted here. Each bar represents mean ± SE (*n* = 3). Means followed by the same letter(s) do not differ by LSD test at 5% probability level (α = 0.05). The colors are just visual ques for readers to quickly distinguish between control and most stressed treatment.

The innate defence system of lemongrass exhibited pronounced antioxidative activities to resist salt-induced oxidative shock. However, it is apparent from Figures 7A–C that the lemongrass antioxidative system was not enough to tolerate higher salinity levels (particularly 160 and 240 mM). Thus, plants suffered the severest and had the lowest growth and development during these treatments. The antioxidant system was most intensified during NaCl 240 mM with an increase of about 47, 43, and 47% in CAT, POX, and SOD activities, respectively, over the control.

Parallel to enzymatic antioxidants, the osmolyte content rose with increasing salt severity. PRO, a key osmolyte, was quantified to appraise osmoprotection during salinity stress in the present experiment. Although each NaCl treatment produced magnified PRO content, the highest upgradation was brought by NaCl 240 mM application with an increase of about 122% in PRO content over the control (Figure 7D).

### Essential Oil Machinery Under Salinity

**FIGURE 8:**
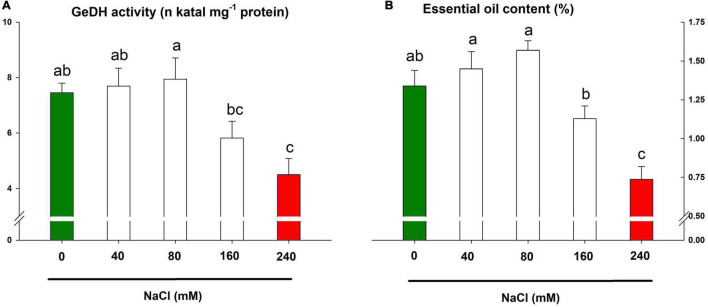
Lemongrass essential oil machinery under salinity stress. The impact of different salt concentrations (0, 40, 80, 160, and 240 mM) was observed on geraniol dehydrogenase (GeDH) activity **(A)** and essential oil content **(B)** of lemongrass plants. Each bar represents mean ± SE (*n* = 3). Means followed by the same letter(s) do not differ by LSD test at 5% probability level (α = 0.05). The colors are just visual ques for readers to quickly distinguish between control and most stressed treatment.

The activity of GeDH, a key enzyme of the essential oil biosynthetic pathway in lemongrass, exhibited a differential response to saline treatments in comparison to earlier mentioned parameters. The salt concentrations of 40 and 80 mM slightly upregulated the enzyme activity by about 3 and 7%, respectively (Figure 8A). The upregulated enzyme increased essential oil content by about 8 and 17% (Figure 8B). Nevertheless, the higher salt concentrations (160 and 240 mM) reduced the enzyme activity by about 22 and 40%, respectively. The heavy restriction of NaCl 240 mM over the enzymatic profile pointed to the salt severity on the essential oil biosynthesis in lemongrass plants (Figures 8A,B).

This was evident with the drop in essential oil content with salt severity. The reduced photosynthesis, development, and enzymatic activity compromised oil productivity in lemongrass. Therefore, the bottom values were obtained with NaCl 240 mM when it perished oil content by about 45% over the control.

**FIGURE 9:**
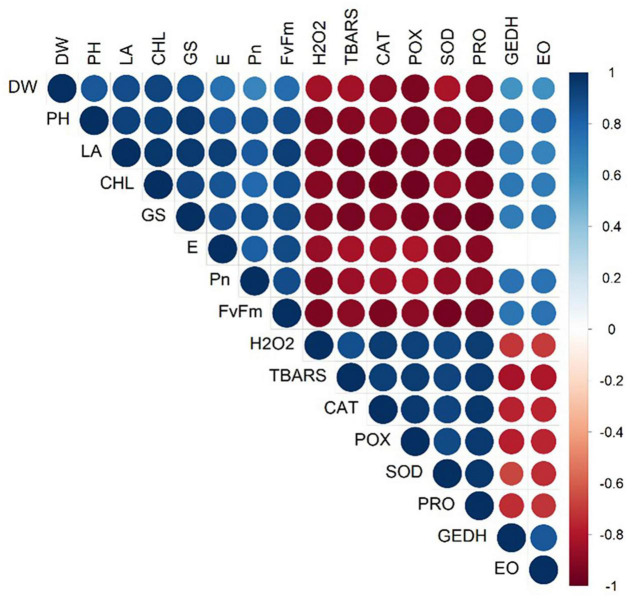
Pearson correlation index heatmap of all tested parameters under different saline regimes (0, 40, 80, 160, and 240 mM of NaCl). The positive correlation is shown with blue colour while the red colour represents a negative correlation. Non-significant correlations at α = 0.05 are excluded from the heatmap. DW, dry weight; PH, plant height; LA, leaf area; CHL, chlorophyll content; GS, stomatal conductance; E, transpiration rate; Pn, net photosynthetic rate; Fv/Fm, quantum yield; H_2_O_2_, hydrogen peroxide content; TBARS, thiobarbituric acid reactive substances content; CAT, catalase activity; POX, peroxidase activity; SOD, superoxide dismutase activity; PRO, proline content; GEDH, geraniol dehydrogenase activity; EO, essential oil content.

**FIGURE 10:**
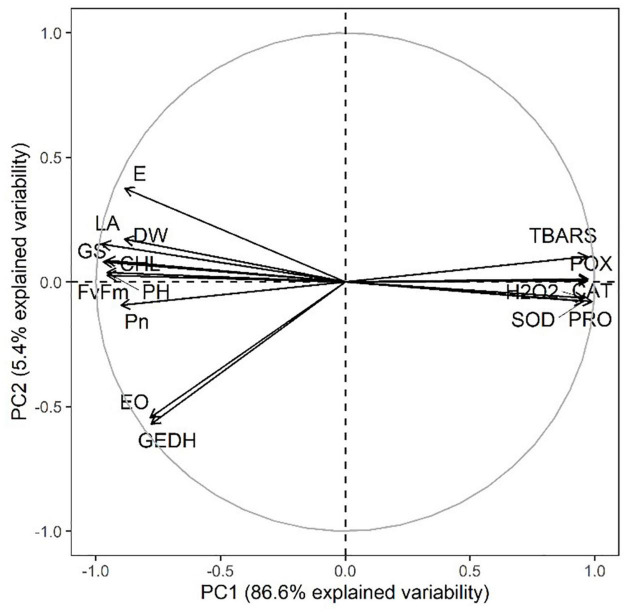
PCA variables factor map of all tested parameters and all five treatments. DW, dry weight; PH, plant height; LA, leaf area; CHL, chlorophyll content; GS, stomatal conductance; E, transpiration rate; Pn, net photosynthetic rate; Fv/Fm, quantum yield; H_2_O_2_, hydrogen peroxide content; TBARS, thiobarbituric acid reactive substances content; CAT, catalase activity; POX, peroxidase activity; SOD, superoxide dismutase activity; PRO, proline content; GEDH, geraniol dehydrogenase activity; EO, essential oil content.

**FIGURE 11:**
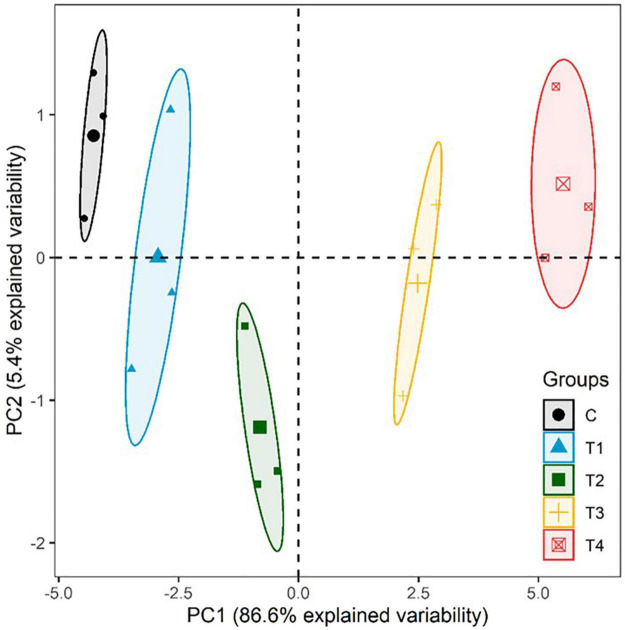
PCA scatter plot with centroids and 95% confidence ellipses. Principal components were derived from all tested parameters and treatments. C, 0 mM NaCl; T1, 40 mM NaCl; T2, 80 mM NaCl; T3, 160 mM NaCl; T4, 240 mM NaCl.

Moreover, a strong correlation was observed among the studied parameters pertaining to the growth, development, and yield of lemongrass plants during all the five salt regimes. A heatmap (Figure 9) was drawn based on Pearson correlation values. All correlation pairings were significant (α = 0.05) except transpiration with geraniol dehydrogenase activity and transpiration with essential oil content. The correlation matrix revealed a strong dependency of lemongrass photosynthetic machinery upon the innate defence system (CAT, POX, SOD, and PRO) of the plant. Moreover, geraniol dehydrogenase activity and essential oil content correlate positively with photosynthetic machinery and negatively with the antioxidant defence system. Furthermore, principal component analysis (PCA) for the various parameters of growth, development, and productivity was carried out. The first two principal components captured 92% of total variability, thus further principal components were dismissed. PCA results showed similar covariance between the traits as shown in correlation analysis (Figure 10). The 95% confidence ellipses within the PCA scatterplot showed that there was no overlap between the treatments based on their overall performance (Figure 11). The treatment exposed to 80 mM (T2) is the only treatment entirely located in the third quadrant of the scatter plot (Figure 11). This position is driven by the high activity of GeDH, and high essential oil content coupled with high photosynthetic performance and growth (Figure 10).

## Discussion

Soil salinity is an acute threat that is continuously claiming irrigable lands around the globe (Qadir et al., 2014). Nevertheless, the damaging effects of salinity can vary depending on its concentration and plant adaptability (Munns and Tester, 2008). Considering a growing market for lemongrass and its essential oil (Haque et al., 2018; Mukarram et al., 2021a,b), the present experiment traces the salt-sensitivity of lemongrass and the efficacy of its defence system against salinity.

### Lemongrass Growth and Salinity

Lemongrass growth was determined in terms of plant height, dry weight, and leaf area. All the salt treatments (40, 80, 160, and 240 mM of NaCl) reduced plant height, dry weight, and leaf area in a dose-dependent manner where the highest salt concentration posed the severest damage. Although the damage occurred in the height and dry weight of the plant at slightly and moderate salinity, the reduction was below 20%. Moderate resistance of lemongrass under salinity stress reflected in stable biomass production was also observed by Ullah et al. (2020). However, with the advent of high and extreme salinity, the height and dry weight of the plant was restricted up to 50%. Similar implications of salinity were observed in many other crops including rice (Kibria et al., 2017), wheat (Saddiq et al., 2021), barley (Zeeshan et al., 2020), maize (Hessini et al., 2019), tomato (Diouf et al., 2018), and potato (Chourasia et al., 2021).

### Salt Implications on Lemongrass Physiology and Its Feedback Mechanism

Salt stress can restrict photosynthesis and associated phenomena that stunts growth and plant development. Photosynthetic retardation is supported by multiple stomatal and non-stomatal restrictions (Kiani-Pouya et al., 2020; Pan et al., 2020). It was noticed that lemongrass, like many other glycophytes, closed their stomata during high (NaCl 160 mM) and extreme (NaCl 240 mM) salinity as a feedback mechanism to minimise the transpiration loss. Nevertheless, it seems pertinent that extended stomatal closure would reduce carbon dioxide intake and, subsequently, carbon assimilation. Similar implications were observed in the present experiment where growing salinity menaced P_*N*_ and E in lemongrass plants. Furthermore, salt-affected lemongrass plants might have higher chlorophyllase activity inhibiting chlorophyll biosynthesis and altering chloroplast ultrastructure through oxidative peroxidation (Flexas et al., 2004; Chaves et al., 2009; Gupta and Pandey, 2020). Further, higher ROS content could also have detrimental effects on lemongrass photosynthetic machinery through an intricate signalling pathway (Foyer, 2018). This might have resulted in reduced chlorophyll content and chlorophyll fluorescence as was observed during different salt regimes.

Increasing salt concentrations in the rhizosphere can limit water and mineral uptake and create an osmotic imbalance. The plant chooses its survival over the growth during inefficient water and nutrient uptake. To achieve osmotic balance, plants direct the energy acquired (from nutrients, water, and photosynthates) to accelerate the production of osmolytes including proline, soluble sugars, polyamines, etc.

Additionally, a general trend of elicitation was noticed in CAT, POX, and SOD activities along with PRO content. The upregulated antioxidant profile and osmolyte during stress is a common defence mechanism in many plants to render enhanced protection against stress-induced oxidative and osmotic damage (Das and Roychoudhury, 2014; Foyer, 2018; Noctor et al., 2018). In addition to salinity stress, higher CAT activity in Lemongrass was also observed during drought (Mirzaei et al., 2020), heavy metal stress (Patra et al., 2019), and seasonal heat (Aziz et al., 2014). Moreover, lemongrass exposed to soil salinity stress increased its SOD activity (Rehman et al., 2022). Similarly, an increase in PRO content in lemongrass was observed under heavy metal stress and drought (Patra et al., 2019; Mirzaei et al., 2020).

### Salinity and Essential Oil Biosynthesis

Since lemongrass oil has antioxidant and antibiological activities, its oil concentration usually rises as a defence mechanism at the onset of a stressful environment (Singh and Anwar, 1985; Mirzaei et al., 2020; Mukarram et al., 2021b). A similar trend was observed during slightly (40 mM) and moderately (NaCl 80 mM) saline treatments even though the differences were not significant. However, at higher salt concentrations (NaCl 160 and 240 mM), LEO machinery might have been exhausted, as evident by the noticeable decrease in lemongrass oil over the control. Similarly, exposure of lemongrass to 150 mM NaCl led to a significant reduction of essential oil content in two cultivars (Idrees et al., 2011). The transcripts related to LEO biosynthesis such as *CfADH1*, *CfADH2a-b*, *CfAAT3*, and *CfALDH*, are responsible for GeDH activity (Meena et al., 2016). Since the GeDH enzyme influences the essential oil biosynthesis in lemongrass, it is feasible to assume these transcripts were downregulated under increased salinity resulting in reduced GeDH activity as we observed with 160 and 240 mM of NaCl. The downgraded enzyme activity can relate to the decreased essential oil content obtained in the present study during the extreme salt regime. However, the essential oil productivity during moderate salinity suggests a viable LEO machinery.

Figure 2 presents the comprehensive understanding of the salt-sensitivity of lemongrass plants devised during the present study. The salt concentration of 80 mM or below imposes photosynthetic restrictions in the plant, however, the closing of stomata, antioxidant activity, and essential oil production are elicited. Lower NaCl concentrations (≤80 mM) induce oxidative stress through ROS accumulation, however, lemongrass antioxidants are also overexpressed at this point for ROS scavenging. This discourages further stress build-up and aids cellular homoeostasis (Figure 2A). On the other hand, higher salinity (≥NaCl 160 mM) compromises photosynthetic assembly and essential oil biosynthesis which result in reduced photosynthates and oil production. Furthermore, extremely low transpiration rate and stomatal conductance seem to have active participation in the salt-induced productivity loss. Although antioxidants were highly active at higher salt concentrations, the accumulated H_2_O_2_ and TBARS content suggest an inefficient defence system in lemongrass during higher salt concentrations (Figure 2B).

## Conclusion

The acute threat of salinity stress is ever-present. Depending on the severity, it poses a reduction in crop growth, development, and productivity, or plant death. Further, different crops respond to salinity differently as a product of their susceptibility. Given the commercial importance of lemongrass, the present experiment was conducted to explore the susceptibility level of lemongrass plants to different salt severities. The result of the study suggests that the lemongrass is a “moderately salt-sensitive” crop. Metabolomic approaches revealed that it can maintain growth and essential oil synthesis under moderate salinity stress (NaCl 80 mM) via upregulation in ROS and antioxidants metabolism. Nevertheless, higher salinity stress (≥NaCl 160 mM) inhibits photosynthesis due to PSII retardation and lowers chlorophyll regeneration in addition to restricting stomatal conductance. Furthermore, the innate defence system of the plant comprising CAT, POX, and SOD antioxidants and osmolyte PRO also struggles to render an efficient antioxidative defence and osmoprotection amidst ROS overaccumulation. This leads to significantly lower growth and essential oil accumulation. Further, the authors suggest the lemongrass for reclamation of saline lands considering that the other members of the Poaceae family are more sensitive to salinity and cannot grow well in such areas. The salinity tolerance threshold of major crops such as rice (30 mM), wheat (60 mM), barley (60 mM), sorghum (65–70 mM), sugarcane (15–20 mM), and maize (15–20 mM) is significantly lower than the lemongrass (80 mM) (Maas and Grattan, 1999). Additionally, the lemongrass holds a special reference to the Indian economy given the exponential growth in lemongrass export (>1250%) within the past two decades (Mukarram et al., 2021b). Therefore, further studies could be relevant in extending the cultivation of lemongrass crops in reclaiming the salt-affected lands (≤80 mM).

## Data Availability Statement

The raw data supporting the conclusions of this article will be made available by the authors, without undue reservation.

## Author Contributions

MK: conceptualisation, validation, supervision, and project administration. MM and MK: methodology. MM and PP: software and formal analysis. MM and AZ: investigation. DK and MK: resources, writing – review and editing, visualisation, and funding acquisition. MM, AZ, and PP: data curation and writing – original draft preparation. All authors have read and agreed to the published version of the manuscript.

## Conflict of Interest

The authors declare that the research was conducted in the absence of any commercial or financial relationships that could be construed as a potential conflict of interest.

## Publisher’s Note

All claims expressed in this article are solely those of the authors and do not necessarily represent those of their affiliated organizations, or those of the publisher, the editors and the reviewers. Any product that may be evaluated in this article, or claim that may be made by its manufacturer, is not guaranteed or endorsed by the publisher.
